# Continuance Intention in Online Food Delivery Platforms in Colombia: Extending UTAUT2 with Delivery Trust and Food Quality Perception

**DOI:** 10.3390/foods15142430

**Published:** 2026-07-08

**Authors:** Andrés García-Umaña, Jorge Bernal-Peralta, Gabriel Estuardo Cevallos Uve, Adela Connie Alcívar Chávez, Évelyn Fernanda Córdoba, Vagner Beserra

**Affiliations:** 1 Facultad de Administración y Economía, Escuela de Diseño e Innovación Tecnológica, Universidad de Tarapacá, Arica 1000000, Chile; vagner.beserra@gmail.com; 2 Facultad de Administración y Economía, Escuela de Administración y Negocios, Universidad de Tarapacá, Arica 1000000, Chile; jbernal@academicos.uta.cl; 3 Instituto Superior Tecnológico Tsa’chila, Santo Domingo de los Tsáchilas 230102, Ecuador; gecevallos@gmail.com; 4 Facultad de Educación, Turismo, Arte y Humanidades, Carrera Educación, Universidad Laica Eloy Alfaro de Manabí (ULEAM), El Carmen 130201, Ecuador; adela.alcivar@uleam.edu.ec; 5 Independent Researcher, Cali 760000, Colombia; evelyncordobap@gmail.com

**Keywords:** online food delivery, continuance intention, extended UTAUT2, consumer behavior, emerging markets, structural equation modeling, delivery trust, food quality perception

## Abstract

Online food delivery (OFD) platforms have become embedded in everyday consumption, yet the determinants of continued use in emerging Latin American markets remain underexplored. This study examines continuance intention toward OFD applications in Colombia using an extended UTAUT2 model (LAE-UTAUT2) that incorporates two domain-specific constructs, Delivery Trust and Food Quality Perception. Cross-sectional survey data from 2130 active users were analyzed through covariance-based structural equation modeling, with confirmatory factor analysis, reliability and validity assessment (CR, AVE, HTMT) and a multi-procedure common-method-bias check. The direct-effects model explained 56.0% of the variance in continuance intention. Performance Expectancy was the strongest predictor, followed by Hedonic Motivation, Food Quality Perception, Delivery Trust, Social Influence, and Effort Expectancy, whereas Facilitating Conditions were non-significant. Notably, the two domain-specific constructs outranked two established UTAUT2 predictors, and the pattern of effects remained stable across age, gender, and experience subgroups and among habitual users. Income, included as a control, showed no detectable independent effect under an unadjusted bracket measure. The findings position experiential quality and fulfillment trust as central correlates of OFD loyalty in emerging markets, though the cross-sectional, self-reported, and reduced-form design warrants cautious, non-causal interpretation.

## 1. Introduction

The growth of online food delivery (OFD) platforms has transformed the way consumers access food products and gastronomic services. Although this type of platform expanded sharply during the post-pandemic period, its use has since become consolidated as a habitual practice within the digital consumption ecosystem [1,2]. Globally, the OFD market maintains a trajectory of sustained growth, driven by smartphone penetration, urbanization, and the normalization of digital consumer services [3,4]. In this context, a relevant question for consumer behavior research is which factors explain why users continue to use these platforms beyond the initial adoption stage.

The technology acceptance literature has widely employed models such as the TAM, TPB, ECT, and UTAUT2 to explain the adoption and continued use of digital technologies [5,6,7,8]. Among them, UTAUT2 constitutes one of the most integrative frameworks for analyzing consumer behavior toward everyday-use technologies, as it incorporates utilitarian, social, facilitating, and hedonic dimensions [6]. This framework has been applied in prior studies on OFD platforms across different geographical contexts, proving useful for explaining usage and continuance intention [1,9,10,11]. In the specific context of OFD platforms, however, continued use does not depend solely on the digital interface or on ease of use but also on service-specific elements, such as trust in the delivery and the perceived quality of the food received [12,13,14,15].

This distinction is particularly important because continuance intention differs conceptually from adoption intention. Whereas initial adoption tends to be based on prior expectations, continuance is formed from accumulated experiences, the confirmation of expectations, and the evaluation of service performance [7,16,17]. On OFD platforms, such experiences include both digital components and physical and logistical elements: delivery punctuality, order accuracy, the condition of the food, temperature, presentation, and responsiveness to service failures [1,12,13]. For this reason, general technology acceptance models require contextual adaptations that better capture the hybrid nature of this type of service.

In Latin America, and particularly in Colombia, research on the continued use of OFD platforms remains limited compared with regions where the literature on technology acceptance and digital services is more consolidated [9,10,17,18]. The Colombian context offers a relevant scenario owing to the expansion of platforms such as Rappi, PedidosYa, and Uber Eats, as well as the coexistence of users with different income levels, digital experience, and frequency of use [6,19]. Although prior regional evidence on the use of food delivery platforms exists, the available studies remain scarce and, in some cases, rely on smaller samples or specific local contexts [20]. These conditions make it necessary to examine whether the classic UTAUT2 determinants retain their explanatory capacity and whether OFD domain-specific constructs, such as Delivery Trust and Food Quality Perception, provide additional evidence for understanding continuance intention.

In response to these gaps, the present study proposes a contextual extension of UTAUT2 to analyze continuance intention toward OFD platforms in Colombia. The model incorporates five core UTAUT2 constructs, namely Performance Expectancy, Effort Expectancy, Social Influence, Facilitating Conditions, and Hedonic Motivation [6], together with two OFD context-specific constructs: Delivery Trust and Food Quality Perception. Delivery Trust is grounded in the literature on trust in electronic commerce and the reduction in uncertainty in digital transactions [21,22,23], whereas Food Quality Perception draws on the service-quality tradition and on information-systems success models that link perceived quality, satisfaction, and continued use [14,15]. Based on a sample of 2130 active OFD platform users, the study estimates a structural equation model that allows the measurement model and the structural model to be evaluated simultaneously. Specifically, the study is organized in three stages: the first stage assesses the reliability and validity of the measurement model through confirmatory factor analysis; the second stage estimates, by means of structural equation modeling, the effects of the UTAUT2 constructs and the OFD context-specific constructs on continuance intention; and the third stage tests the robustness of the main findings by incorporating demographic and usage-related variables as controls.

## 2. Literature Review

### 2.1. From Technology Acceptance to Post-Adoption Continuance: Positioning UTAUT2 

Research on consumer behavior in digital services has advanced through successive theoretical refinements rather than a mere accumulation of competing models. The Technology Acceptance Model (TAM) [24] established perceived usefulness and ease of use as the proximal determinants of adoption [3] and was subsequently subsumed into the Unified Theory of Acceptance and Use of Technology (UTAUT), which consolidated rival antecedents into performance expectancy, effort expectancy, social influence, and facilitating conditions [25]. UTAUT2 extended this synthesis to consumer settings by incorporating hedonic motivation, price value, and habit, and it reports the highest explanatory power among acceptance frameworks for everyday consumer technologies [6]. Rather than treating these models as chronological alternatives, the present study adopts UTAUT2 as its theoretical spine for three reasons: it is the most parsimonious integrative framework that simultaneously accommodates utilitarian, social, facilitating, and hedonic motivations; it has been validated specifically in OFD contexts [9,10,13,17,18]; and its modular structure can be selectively recalibrated to incorporate domain-specific determinants without forfeiting its nomological coherence.

A central conceptual distinction underpins this choice. Adoption and continuance are not governed by the same mechanisms: initial adoption rests on pre-use expectations, whereas continuance is a post-adoption judgment shaped by confirmed expectations and accumulated experience, as formalized in Expectation-Confirmation Theory (ECT) [7] and its integrative extension to information-systems continuance [26]. UTAUT2 does not, on its own, theorize this experiential feedback loop; ECT supplies it. Accordingly, this study frames the dependent construct as continuance intention and interprets the UTAUT2 antecedents as post-experience evaluations rather than anticipatory beliefs, an orientation consistent with the way acceptance constructs behave once usage has become routine [7,26]. 

Evidence from OFD applications of UTAUT2 is, however, less uniform than is frequently acknowledged, and a critical reading reveals systematic divergences rather than a settled consensus. Performance expectancy and hedonic motivation are repeatedly identified as dominant drivers of usage and continuance [1,10,17], yet effort expectancy and facilitating conditions exhibit unstable effects: both tend to lose explanatory weight among experienced users, for whom interface mastery and infrastructure access are no longer differentiating [13,17]. Social influence is similarly contingent, exerting stronger effects in collectivist or socially networked consumption environments and weaker effects elsewhere [11,18]. This heterogeneity motivates a re-examination of the canonical predictors in an under-studied Latin American market, where their configuration has not been empirically established [6,11]. 

Building on this assessment, the proposed model retains the five UTAUT2 antecedents most defensible in a post-adoption service context, namely performance expectancy, effort expectancy, social influence, facilitating conditions, and hedonic motivation, while deliberately substituting price value and habit [6,25]. This decision is theoretical rather than merely pragmatic. Habit, as originally specified within UTAUT2, is conceptually entangled with prior behavior and usage frequency, and therefore risks tautology when both predictor and outcome are usage-based [6]; frequency of use is consequently modeled here as a control variable rather than a latent driver, consistent with the post-adoption logic of information-systems continuance research [7,26]. Price value, in turn, overlaps substantially with the utilitarian benefit–cost appraisal already embodied in performance expectancy once consumption has become habitual [6]. The omission of price value warrants particular justification given the price sensitivity that characterizes emerging-market consumers. Three considerations support its exclusion here. Conceptually, once OFD use has become routine, the cost–benefit appraisal that price value formalizes is largely absorbed into performance expectancy, with which it correlates strongly in consumer settings [6]. Operationally, a price-value construct would conflate platform fees, restaurant pricing, and promotional dynamics that differ across the three platforms sampled (Rappi, PedidosYa, and Uber Eats), introducing measurement heterogeneity that compromises reflective-scale comparability. Empirically, the income-stratified robustness analysis allows price-related socioeconomic variation to be partly absorbed through the income control. A dedicated price-value construct could nonetheless yield additional insight in this market and is identified as a priority for subsequent specifications.

In their place, the model introduces two constructs that capture the hybrid digital-physical nature of OFD [13,17] and are theoretically proximal to continuance in this domain: Delivery Trust, grounded in the e-commerce trust tradition [21,22,23], and Food Quality Perception, grounded in the service-quality and information-systems success literature [14,15]. The resulting framework is thus a focused, domain-calibrated extension of UTAUT2, comparable in spirit to prior context-specific extensions of the model in food delivery research [9,13], rather than a wholesale reconstruction; its contribution lies in this recalibration and its empirical test, not in the conceptual novelty of the added constructs per se [11]. Consistent with this reasoning and with prior OFD evidence on the canonical antecedents [1,9,10,13,17,18], the following hypotheses are proposed:

**H1.**  *Performance Expectancy positively influences continuance intention toward OFD platforms.*

**H2.**  *Effort Expectancy positively influences continuance intention toward OFD platforms.*

**H3.**  *Social Influence positively influences continuance intention toward OFD platforms.*

**H4.**  *Facilitating Conditions positively influence continuance intention toward OFD platforms.*

**H5.**  *Hedonic Motivation positively influences continuance intention toward OFD platforms.*

### 2.2. Delivery Trust: Relational Risk in a Hybrid Service

Trust has long been theorized as the principal mechanism through which consumers manage uncertainty and perceived risk in digital exchange [21,22]. Ref. [21] demonstrate that trust complements technology-acceptance beliefs by attenuating the social uncertainty inherent in online transactions, while ref. [22] positions trust and perceived risk as co-determinants of behavioral intention in electronic commerce. Ref. [23] further show that trust operates not only directly but also by shaping perceived value and risk, reinforcing its role in sustained engagement.

Most OFD research, however, operationalizes trust at the platform or vendor level and rarely isolates the fulfillment stage, where the hybrid character of the service is most exposed. OFD departs from purely digital commerce because the transaction is completed offline, through a physical handoff vulnerable to lateness, inaccuracy, and unresolved failures [13,17]. Delivery Trust is therefore defined narrowly here as the consumer’s confidence that the platform will fulfill orders accurately, punctually, and with effective recovery when problems arise. This delivery-specific conceptualization is particularly consequential in emerging markets, where weaker consumer-protection institutions and higher perceived transactional risk raise the behavioral premium on reliable fulfillment [27,28]. Treating Delivery Trust as a distinct determinant thus extends the OFD trust literature beyond generic platform trust toward the operational reliability that conditions continuance.

**H6.**  *Delivery Trust positively influences continuance intention toward OFD platforms.*

### 2.3. Disentangling Service Quality from Food Quality Perception

Perceived quality is conventionally theorized as the gap between expected and perceived performance, as formalized in SERVQUAL [15] and echoed in the service-quality dimension of the DeLone and McLean information-systems success model [14]. In digital services, this tradition captures the quality of the service encounter and the system: responsiveness, reliability of the interface, assurance, and the handling of the order process. Applied to OFD, these frameworks describe the quality of the platform’s service delivery.

A conceptual boundary that prior OFD studies frequently blur must therefore be drawn explicitly. Service quality, in the SERVQUAL [15] and IS-success [14] sense, concerns the process through which the order is mediated and delivered, encompassing responsiveness, reliability, and assurance in the service encounter [15]. Food Quality Perception, by contrast, concerns the outcome attributes of the physical product that reaches the consumer, namely temperature retention, freshness, presentation integrity, and taste fidelity after transit [20]. The two are empirically correlated yet conceptually independent: a punctual, courteous, and technically flawless delivery (high service quality) can still yield a cold or damaged meal (low food quality), and the reverse is equally possible. This distinction matters because perceived quality drives satisfaction and loyalty only insofar as it reflects the dimensions consumers actually evaluate [17], and in OFD the physical outcome constitutes a salient evaluative dimension not represented in process-oriented service-quality models [20]. Food Quality Perception is consequently not a relabeling of service quality but the tangible, consumption-stage extension of quality that is unique to food-bearing logistics and that standard acceptance [6] and service-quality [14,15] constructs do not capture.

Because perceived quality is a well-established antecedent of satisfaction and loyalty [17], and because food quality has begun to surface as a continuance predictor in OFD research [20], its omission from acceptance-based models plausibly understates the experiential basis of loyalty. The argument is sharper in emerging markets, where greater variability in product condition during delivery increases the diagnostic weight that consumers assign to the physical outcome [20]. Therefore, the following research hypothesis is proposed.

**H7.**  *Food Quality Perception positively influences continuance intention toward OFD platforms.*

### 2.4. The LAE-UTAUT2 Framework: Theoretical Integration

No single theory accounts for the full behavioral architecture of OFD continuance, and the value of an integrative model lies in assigning each source framework a non-redundant function rather than juxtaposing them. UTAUT2 [6] contributes the technological and motivational antecedents; ECT [7,26] supplies the post-adoption logic that converts those antecedents into continuance judgments; the trust tradition [21,22,23] adds the relational dimension of fulfillment risk; and the SERVQUAL and IS-success lineage [14,15] grounds the outcome-quality dimension. The Theory of Planned Behavior [8] and the diffusion-of-innovations perspective [29,30] provide complementary scaffolding, linking intention to attitudes, norms, and perceived control and to perceptions of relative advantage and compatibility, without introducing constructs that overlap with those already specified. Table 1 summarizes how each framework contributes a distinct theoretical function to the proposed model.

Integrated in this manner, the Latin American Extended UTAUT2 (LAE-UTAUT2) is best understood not as the invention of new constructs, since Delivery Trust and Food Quality Perception are recognizable extensions of established trust and quality traditions, but as a deliberate, domain-calibrated configuration whose contribution is threefold: it embeds two OFD-salient determinants within a coherent continuance architecture; it tests whether these determinants rival the canonical UTAUT2 antecedents in an under-studied emerging market; and it examines the structural stability of the resulting model across income strata. This specification estimates direct effects on continuance intention within a covariance-based structural equation modeling framework and is therefore best understood as a reduced-form model. Although Expectation-Confirmation Theory posits that post-adoption evaluations influence continuance partly through satisfaction [7,26], the present model estimates the net association of each determinant with continuance without incorporating satisfaction as an intervening construct; the reported coefficients should accordingly be read as composite effects that subsume any mediation operating through satisfaction or related evaluative states. Modeling these sequential mechanisms explicitly—particularly satisfaction with service fulfillment in the OFD context—remains theoretically plausible and is identified as the principal extension for subsequent confirmatory and longitudinal research (Section 6.2).

## 3. Method

### 3.1. Research Design and Sampling

A cross-sectional quantitative design was employed, consistent with the methodological tradition used in the validation and extension of the Unified Theory of Acceptance and Use of Technology 2 (UTAUT2) in contexts of adoption and continued use of digital technologies [6]. The target population comprised active users of online food delivery (OFD) applications in Colombia who reported having used one of these platforms during the three months prior to data collection.

Data were collected between January and August 2025 through a structured questionnaire administered via a multimodal data-collection strategy. The instrument was distributed both digitally, through online platforms, social networks, and virtual communities of OFD application users, and through interviewer-assisted administration by previously trained surveyors, who supported the in-person application of the questionnaire across different consumption settings. This strategy made it possible to broaden the geographical coverage of the research, increase the diversity of participants, and reduce the limitations associated with the exclusive use of online surveys.

In order to capture a heterogeneous sample representative of different consumer profiles, the assisted administration was conducted in several Colombian cities: Bogotá, Medellín, Cali, Barranquilla, and Bucaramanga. The combination of collection modalities facilitated the participation of users belonging to different age groups, income levels, and digital consumption patterns, contributing to greater sample variability and strengthening the external validity of the results.

Before completing the questionnaire, all participants were informed about the academic objectives of the research, the voluntary nature of their participation, and the guarantees of confidentiality and anonymity of the information provided. In addition, a filter question was included to verify that respondents had used an online food delivery application during the three months prior to the study, thereby ensuring the relevance of the responses obtained with respect to the phenomenon under analysis.

As a result of the data-collection process, 2130 valid responses were obtained. All observations contained complete information for the indicators included in the research model, and the full set of cases was therefore retained for the subsequent analyses. The use of a large sample increases the precision of the estimates and the stability of the parameters obtained through structural equation modeling. Likewise, the size of the sample, the multimodal data-collection strategy, and the coverage of multiple cities are considered to strengthen the empirical robustness of the study and the generalizability of the findings within the national context.

### 3.2. Measurement Instrument and Measurement Model Assessment 

All constructs were operationalized through multi-item reflective scales measured on a seven-point Likert scale (1 = strongly disagree; 7 = strongly agree). The UTAUT2 constructs—Performance Expectancy, Effort Expectancy, Social Influence, Facilitating Conditions, and Hedonic Motivation—were adapted from [6] and from prior research conducted in the context of online food delivery (OFD) applications [9,10]. Continuance Intention was measured following Bhattacherjee’s expectation-confirmation model [19] and studies specifically addressing continued use on OFD platforms [1]. Delivery Trust was grounded in the literature on trust in electronic commerce [21,22,23], whereas Food Quality Perception was constructed on the basis of the service-quality literature [15] and information-systems success [14], considering attributes such as freshness, presentation, temperature, and the condition of the delivered product. Consistent with the post-adoption logic set out in Section 2.1, price value and habit were not modeled as latent predictors; usage frequency was instead retained as a control variable to avoid the predictor–outcome tautology that a usage-based habit construct would introduce [6,7,26]. All items were adapted to reflect post-adoption evaluations and continuance-oriented behaviors.

To ensure the semantic equivalence of the scales, the questionnaire was translated into Spanish by two bilingual experts and subsequently subjected to a back-translation procedure carried out by an independent translator. The psychometric quality of the instrument was assessed through Confirmatory Factor Analysis (CFA) within the Covariance-Based Structural Equation Modeling (CB-SEM) approach, examining standardized factor loadings, internal consistency through Cronbach’s alpha and Composite Reliability (CR), convergent validity through the Average Variance Extracted (AVE), and overall model fit through the χ^2^, Comparative Fit Index (CFI), Tucker–Lewis Index (TLI), and Root Mean Square Error of Approximation (RMSEA) indices [31]. 

Because the data were collected through self-reported measures at a single point in time, potential common method bias (CMB) [32] was assessed using multiple diagnostic procedures. First, the fit of a single-factor model was compared with that of the multidimensional theoretical model. A substantially poorer fit of the single-factor specification was interpreted as evidence that the covariance among the observed indicators could not be explained by a single underlying factor.

Because the questionnaire did not include a theoretically unrelated marker variable, a marker-variable approach could not be implemented. Therefore, the assessment was complemented by estimating a common latent factor (CLF) sensitivity model. The results of this procedure were interpreted cautiously, as the inclusion of a generic method factor in the absence of a marker variable may capture not only method-related variance but also part of the constructs’ substantive variance, potentially leading to an overestimation of common method effects.

### 3.3. Structural Model Analysis

Once the measurement model had been validated, the structural model was estimated through CB-SEM to examine the effects of Performance Expectancy, Effort Expectancy, Social Influence, Facilitating Conditions, Hedonic Motivation, Delivery Trust, and Food Quality Perception on continuance intention in the use of OFD applications. The evaluation considered both the statistical significance of the standardized path coefficients and the model’s explanatory capacity, assessed through the coefficient of determination (R^2^). Information criteria, including the Akaike Information Criterion (AIC) and the Bayesian Information Criterion (BIC), were also reported to support model comparison and parsimony assessment [33].

Because all structural paths linking the seven exogenous constructs to continuance intention were freely estimated and the exogenous constructs were allowed to covary, the proposed model constitutes a recursive and structurally saturated specification. Consequently, it reproduces the same model-implied covariance matrix as the validated measurement model, rendering both specifications statistically equivalent in terms of global fit. As a result, identical values of degrees of freedom, χ^2^, CFI, TLI, RMSEA, AIC, and BIC are obtained. No correlated error terms were released during estimation; therefore, the reported fit indices reflect the originally specified measurement structure rather than post hoc model respecification.

To further assess the stability and robustness of the findings, several complementary analyses were performed. Approximate multigroup SEM comparisons were conducted across age, gender, and OFD experience categories to explore potential subgroup heterogeneity. A rival-model comparison was then performed to determine whether Performance Expectancy and Continuance Intention represented empirically distinct latent constructs. Given that the questionnaire did not include a theoretically unrelated marker variable, a common latent factor sensitivity model was additionally estimated as a diagnostic procedure for potential common method bias. The structural model was also re-estimated using a restricted subsample of habitual users to evaluate the consistency of the results across different usage profiles. Finally, Cohen’s f^2^ effect sizes were calculated by sequentially removing each predictor from the full model, thereby examining the practical magnitude of each construct’s contribution to the explanation of continuance intention.

## 4. Results

### 4.1. Sample Demographic

Table 2 presents the demographic and behavioral profile of the participants. The sample was composed predominantly of women (53.2%) and men (42.5%), while a smaller proportion identified as non-binary or another gender identity (2.0%) or preferred not to disclose this information (2.2%). With regard to age, the most representative group corresponded to participants between 25 and 34 years of age (38.2%), followed by those between 18 and 24 years (28.8%), which reflects a substantial presence of young consumers within the online food delivery application market in Colombia.

From a geographical perspective, participants were concentrated in the country’s main cities: Bogotá (36.9%), Medellín (23.1%), Cali (16.3%), and Barranquilla (11.0%), although participation was also recorded from users in other cities (12.6%). The sample likewise exhibited a relatively high educational level, as 64.2% of respondents held an undergraduate (40.5%) or postgraduate (23.7%) university degree. Regarding employment status, the sample was dominated by employed individuals (38.7%), students (24.4%), and self-employed or freelance workers (19.7%), reflecting a diverse representation of economically active consumers.

In relation to income, the lower-middle-income segment constituted the largest group (32.2%), followed by the upper-middle-income segment (26.7%), evidencing adequate socioeconomic diversity among participants. As for platform preference, Rappi was identified as the main food delivery application by 46.7% of respondents, followed by users who reported regularly using more than one application (21.3%), PedidosYa (18.0%), and Uber Eats (14.0%), results consistent with the market leadership that Rappi maintains in Colombia.

Finally, the sample demonstrated a high level of familiarity with online food delivery services. More than three quarters of the participants (78.0%) reported at least one year of experience using OFD applications, while 39.3% indicated more than three years of experience. Likewise, frequent usage patterns were observed, as 62.1% of respondents reported using these platforms at least once a week. Taken together, these characteristics suggest that the sample is composed primarily of experienced and habitual users, capable of providing informed evaluations of the factors that influence continuance intention in the use of online food delivery applications.

### 4.2. Descriptive Statistics and Reliability

Table 3 presents the descriptive statistics and reliability coefficients for the study constructs. All Cronbach’s alpha values exceeded the recommended threshold of 0.70, ranging from α = 0.763 for Hedonic Motivation (HM) to α = 0.833 for Continuance Intention (CI), indicating satisfactory internal consistency. Effort Expectancy showed the highest mean score (M = 5.840, SD = 0.793), suggesting that respondents generally perceived OFD applications as easy to use. Performance Expectancy (M = 5.566, SD = 0.874) and Facilitating Conditions (M = 5.548, SD = 0.899) also displayed relatively high mean values. In contrast, Delivery Trust (M = 4.702, SD = 1.049) and Food Quality Perception (M = 4.656, SD = 1.024) reported the lowest mean scores and comparatively higher dispersion, indicating greater variability in users’ evaluations of logistical trust and perceived food quality. Finally, Continuance Intention showed a positive but non-ceiling-level mean score (M = 5.298, SD = 0.982), suggesting that users generally intend to continue using OFD platforms, although loyalty remains open to variation across user experience dimensions.

### 4.3. Measurement Model Assessment: CFA, Reliability, and Convergent Validity

Table 4 presents the results of the confirmatory factor analysis (CFA) for the measurement model, including standardized factor loadings, composite reliability (CR), and average variance extracted (AVE). All standardized factor loadings were statistically significant (*p* < 0.001) and ranged from 0.690 to 0.767, indicating adequate relationships between the observed indicators and their respective latent constructs, while composite reliability values ranged from 0.763 to 0.833, comfortably exceeding the 0.70 threshold. AVE values ranged from 0.499 to 0.583: Delivery Trust returned an AVE of 0.499, marginally below the conventional 0.50 criterion before rounding, and four further constructs (PE = 0.515, HM = 0.518, EE = 0.540, SI = 0.545) fell within the 0.50–0.55 band. Following Fornell and Larcker [34] and the established guidance that convergent validity may be deemed acceptable when AVE is marginal but composite reliability comfortably exceeds 0.70, these constructs were retained, a decision further supported by the discriminant validity evidence in the HTMT matrix (Table 5; all values ≤ 0.624). The pattern of moderate, homogeneous loadings is consistent with reliable but breadth-oriented reflective measures whose items tap somewhat heterogeneous facets of each domain rather than a narrow common core, as is theoretically expected for parsimonious acceptance scales. Overall, these results support the adequacy of the measurement model and provide evidence of convergent validity for the LAE-UTAUT2 constructs, although Delivery Trust is flagged as the construct most in need of refinement.

Table 5 presents the HTMT matrix used to assess discriminant validity among the latent constructs. All HTMT values were below the conservative threshold of 0.85, ranging from 0.270 to 0.624. The highest value was observed between Performance Expectancy and Continuance Intention, which remains well below the recommended cutoff. These results indicate that the constructs are empirically distinguishable and support the discriminant validity of the measurement model.

Given that the highest HTMT value was observed between Performance Expectancy and Continuance Intention (HTMT = 0.624), an additional rival-model comparison was conducted to assess whether both constructs were empirically distinguishable. The two-factor model, in which PE and CI were specified as separate latent variables, showed excellent fit (χ^2^ = 27.661, df = 19, CFI = 0.999, TLI = 0.998, RMSEA = 0.015). In contrast, the one-factor model, in which all PE and CI items loaded on a single latent factor, showed substantially poorer fit (χ^2^ = 1164.300, df = 20, CFI = 0.821, TLI = 0.749, RMSEA = 0.164). The chi-square difference was also substantial (Δχ^2^(1) = 1136.639, *p* < 0.001), supporting the discriminant validity of Performance Expectancy and Continuance Intention.

Table 6 reports the global fit indices for the CFA measurement model, the SEM, and the single-factor model used as a common method bias check. Both the measurement and structural models showed excellent fit, with CFI and TLI values above 0.99 and RMSEA below 0.01. In contrast, the single-factor model showed poor fit (CFI = 0.590, TLI = 0.557, RMSEA = 0.111), indicating that the covariance structure of the data was not adequately explained by a single common factor. These results support the adequacy of the proposed multi-construct model and reduce concerns that common method bias alone accounts for the observed relationships.

As an additional sensitivity check, a common latent factor model was estimated. This model showed excellent fit (χ^2^ = 314.849, df = 294, CFI = 0.999, TLI = 0.999, RMSEA = 0.006), and the explained variance in Continuance Intention remained highly similar to the main SEM (R^2^ = 0.564 versus R^2^ = 0.560). However, because no marker variable was available, this result is interpreted only as a supplementary diagnostic rather than as definitive evidence that common method bias is absent.

### 4.4. Structural Model and Hypothesis Testing

Table 7 presents the SEM structural path estimates for the hypothesized relationships. The model explained approximately 56.0% of the variance in Continuance Intention. Performance Expectancy was the strongest predictor of Continuance Intention (β = 0.311, *p* < 0.001), followed by Hedonic Motivation (β = 0.181, *p* < 0.001), Food Quality Perception (β = 0.176, *p* < 0.001), Delivery Trust (β =0.145, *p* < 0.001), Social Influence (β = 0.138, *p* < 0.001), and Effort Expectancy (β =0.068, *p* = 0.017). Facilitating Conditions did not have a statistically significant effect on Continuance Intention (β = 0.024, *p* = 0.398). Therefore, H1, H2, H3, H5, H6, and H7 were supported, whereas H4 was not supported.

Cohen’s f^2^ values were calculated to assess the incremental contribution of each predictor to the explained variance in Continuance Intention. Performance Expectancy showed the largest incremental effect (f^2^ = 0.084), followed by Food Quality Perception (f^2^ = 0.025), Hedonic Motivation (f^2^ = 0.020), Social Influence (f^2^ = 0.017), and Delivery Trust (f^2^ = 0.014). Effort Expectancy and Facilitating Conditions showed negligible incremental effects (f^2^ ≈ 0.000). These results indicate that the practical contribution of the predictors varies substantially, with Performance Expectancy providing the strongest unique explanatory contribution.

### 4.5. Robustness Analysis with Control Variables

To assess the robustness of the structural model, an additional SEM specification was estimated including demographic and usage-related control variables: gender, age, educational level, income level, frequency of use, and experience using OFD applications. The results remained substantively consistent with the main SEM. Performance Expectancy, Effort Expectancy, Social Influence, Hedonic Motivation, Delivery Trust, and Food Quality Perception continued to show significant positive effects on Continuance Intention, whereas Facilitating Conditions remained non-significant. Among the control variables, only frequency of use showed a statistically significant positive association with Continuance Intention, suggesting that more frequent users tend to report stronger intentions to continue using OFD platforms. Overall, the robustness model supports the stability of the main findings after accounting for key demographic and usage characteristics.

A further sensitivity analysis was conducted by re-estimating the SEM among habitual users only, defined as respondents who used OFD applications at least monthly and had at least six months of experience with these platforms. This restricted sample included 1748 respondents. The model showed excellent fit (χ^2^ = 351.177, df = 322, CFI = 0.998, TLI = 0.998, RMSEA = 0.007) and explained 56.3% of the variance in Continuance Intention, which is highly consistent with the full-sample model (R^2^ = 0.560). The pattern of results remained stable: Performance Expectancy (β = 0.320, *p* < 0.001), Effort Expectancy (β = 0.094, *p* = 0.003), Social Influence (β = 0.133, *p* < 0.001), Hedonic Motivation (β = 0.188, *p* < 0.001), Delivery Trust (β = 0.137, *p* < 0.001), and Food Quality Perception (β = 0.176, *p* < 0.001) remained significant, whereas Facilitating Conditions remained non-significant (β = −0.007, *p* = 0.831). This confirms that the main findings are robust when the analysis is restricted to more active and experienced users.

Approximate multigroup SEM comparisons were also conducted to examine whether the structural paths varied by age, gender, and OFD experience. Age was compared between respondents younger than 35 years and those aged 35 years or older; gender was compared between female and male respondents; and experience was compared between users with less than one year of OFD experience and those with one year or more. No structural path showed statistically significant differences across these subgroup comparisons, with all approximate two-tailed *p*-values above 0.12. Therefore, the effects observed in the main SEM appear broadly stable across age, gender, and experience groups. Respondents identifying as non-binary/other or preferring not to disclose gender were not included in the binary gender comparison because of their smaller cell sizes.

## 5. Discussion

The present study estimated a covariance-based structural equation model (CB-SEM) of continuance intention toward online food delivery (OFD) platforms in Colombia, integrating five UTAUT2 antecedents with two domain-specific constructs, Delivery Trust and Food Quality Perception. The model accounted for 56.0% of the variance in continuance intention, and six of the seven hypothesized paths were supported (H1, H2, H3, H5, H6, and H7), whereas facilitating conditions did not exert a significant effect (H4 not supported). The following sections interpret these findings in relation to prior OFD research, emphasizing the relative magnitude and theoretical meaning of the effects rather than statistical significance alone, given that the large sample (*n* = 2130) renders even modest associations statistically detectable [35].

### 5.1. Performance Expectancy and the Utilitarian Core of Continuance

Performance Expectancy emerged as the strongest predictor of continuance intention (β = 0.311, *p* < 0.001), and its association with the outcome was the highest in the construct matrix (HTMT = 0.624). This dominance is consistent with the theoretical architecture of UTAUT2 [6] and replicates the central role of perceived usefulness reported across OFD studies in diverse markets [1,9,17]. In the Colombian context, the finding suggests that continuance decisions are anchored primarily in the platforms’ perceived capacity to deliver utilitarian value, that is, time savings, convenience, and dependable access to a broad set of options. The pattern indicates that, for experienced users, functional performance and reliability may matter more for retention than incremental interface refinements; this interpretation, however, should be read as a plausible implication rather than a demonstrated causal mechanism, given the cross-sectional design.

### 5.2. The Domain-Specific Constructs: Food Quality Perception and Delivery Trust

The most theoretically salient findings concern the two added constructs. Food Quality Perception (β = 0.176, *p* < 0.001) and Delivery Trust (β = 0.145, *p* < 0.001) were both significant and, notably, exceeded the effects of two UTAUT2 antecedents, namely effort expectancy and facilitating conditions. This ordering supports the central argument that generic acceptance constructs do not fully capture the determinants of continuance in a hybrid digital–physical service [13,17] and that outcome quality and fulfillment reliability carry independent explanatory weight. The result is consistent with emerging OFD evidence that incorporates food quality as a continuance predictor [20] and with the broader trust literature in electronic commerce [21,22,23].

The prominence of Food Quality Perception is theoretically coherent with the experiential nature of food consumption: unlike purely digital services, OFD culminates in a physical product whose temperature, freshness, and presentation may degrade in transit, and whose evaluation feeds directly into satisfaction and loyalty [15,17]. Its discriminant separation from the remaining constructs reinforces that it captures a distinct evaluative dimension rather than a relabeling of service quality [14,15]. Delivery Trust, in turn, reflects the relational dimension of fulfillment risk; its significant effect is consistent with the view that trust mitigates the uncertainty inherent in transactions completed offline [21,22,23], a concern plausibly heightened in markets where consumer-protection mechanisms for digital transactions are still maturing [27,28]. Taken together, these results substantiate the domain-calibrated extension of UTAUT2 proposed here while remaining consistent with the more modest framing that these constructs refine rather than overturn the model [11].

### 5.3. Hedonic Motivation and Social Influence

Hedonic Motivation was the second strongest predictor (β = 0.181, *p* < 0.001), underscoring that the recreational and experiential appeal of OFD platforms contributes to continuance beyond their functional utility. This aligns with the sample’s predominantly young and digitally engaged profile and with prior OFD findings on the role of enjoyment in sustained use [10,17]. Social Influence also exerted a significant, though smaller, effect (β = 0.138, *p* < 0.001), consistent with evidence that interpersonal recommendation and socially mediated discovery shape OFD adoption and continuance [11,18]. Its moderate magnitude suggests that, once usage is established, social pressure operates as a supporting rather than a primary driver of retention.

### 5.4. Effort Expectancy and the Non-Significant Role of Facilitating Conditions

Effort Expectancy showed a small but significant effect (β = 0.068, *p* = 0.017). Its limited magnitude is theoretically coherent with the sample’s high experience level, as most respondents reported more than one year of platform use, an attenuation pattern documented in other experienced-user OFD samples [13,17] and consistent with the notion that ease of use functions as a threshold condition for adoption that loses differentiating power for continuance. Facilitating Conditions, by contrast, did not significantly predict continuance intention (β = 0.024, *p* = 0.398), and H4 was therefore not supported. This null result is plausibly attributable to the urban, digitally connected, and relatively well-educated composition of the sample, for whom enabling resources such as connectivity, devices, and payment infrastructure are largely taken for granted and thus no longer differentiate users [17]. The robustness model corroborated this pattern, with facilitating conditions remaining non-significant after the inclusion of controls.

### 5.5. The Role of Income and Demographic Controls

The robustness analysis incorporating demographic and usage-related controls (Table 8) left the structural findings substantively unchanged, supporting their stability. Among the controls, income level did not exert a detectable independent effect on continuance intention (β = 0.020, *p* = 0.264), indicating that, once the perceptual determinants included in the model are accounted for, income bracket did not explain additional variation in continuance intention under the present specification. This finding is noteworthy in an income-stratified design because it suggests that the determinants of OFD continuance may operate broadly across socioeconomic segments rather than being confined to higher-income users. However, this null result should be interpreted with caution rather than as evidence of genuine equivalence across income groups. Income was operationalized using absolute monthly brackets in Colombian pesos, without adjustment for household size, number of dependents, or purchasing power. Such a relatively coarse operationalization may reduce measurement precision and obscure an underlying association between economic resources and continuance behavior. Accordingly, the finding is better interpreted as the absence of a detectable independent effect under the present measure rather than confirmation that income is immaterial to continuance. We also refrain from interpreting this pattern as evidence of formal structural or measurement invariance, since establishing invariance would require multigroup confirmatory analyses across income segments, which lay beyond the scope of the present estimation. Future research should therefore employ per capita or purchasing-power-adjusted measures of income and formally test invariance across socioeconomic groups to determine whether the determinants of continuance truly operate equivalently across income strata.

Among all controls, only frequency of use was significantly associated with continuance intention (β = 0.137, *p* < 0.001), an expected pattern indicating that more frequent users report stronger continuance intentions. This finding further reinforces the methodological decision to retain usage frequency as a control variable rather than modeling a latent habit construct, thereby avoiding the predictor–outcome tautology that a usage-based habit measure could introduce while still capturing the influence of accumulated experience on continuance intentions.

## 6. Conclusions

### 6.1. Theoretical, Practical, and Social Implications

This study advances the understanding of continuance intention in online food delivery (OFD) platforms in two main ways. First, it extends and validates the UTAUT2 framework in Colombia, an under-researched yet dynamic Latin American OFD market, showing that the canonical architecture benefits from domain-specific extension in emerging-market contexts. Second, by integrating Delivery Trust and Food Quality Perception, it proposes the LAE-UTAUT2 model. Consistent with evidence that these dimensions are already established in OFD research, the contribution lies in their integration and empirical test within a coherent continuance model, in which they outranked effort expectancy and facilitating conditions, rather than in conceptual novelty. The large sample (*n* = 2130) and covariance-based structural equation estimation provide a robust basis for the estimates, which remained stable under demographic and usage-related controls.

The income-stratified sampling ensured representation across economic segments, and no detectable independent effect of income was observed under the unadjusted income measure employed in this study. This finding should be interpreted as descriptive of the present model rather than as evidence of formal structural invariance, which would require multigroup confirmatory testing and remains an important avenue for future research.

For platform operators, the findings offer prioritization cues rather than prescriptive mandates. The primacy of Performance Expectancy points to service consistency, that is, on-time delivery, order accuracy, and reliable performance, as a foundation for retention. The significant effects of Food Quality Perception and Delivery Trust suggest that the physical-product outcome (packaging, thermal preservation, transit-time management) and fulfillment reliability (transparent dispute resolution and visible service commitments) may be especially consequential here, since both rivaled or exceeded the convenience-related predictors. Hedonic Motivation further indicates that discovery-oriented, personalized experiences may sustain engagement among the young, digitally active user base.

At the policy level, although Facilitating Conditions did not significantly predict continuance in this urban, well-educated sample, infrastructure and financial-inclusion initiatives may still matter for extending OFD participation to underserved populations. The salience of Delivery Trust suggests that regulatory attention to digital consumer protection, including order-accuracy guarantees, rider accountability, and dispute resolution, could address the trust concerns identified here and support the sustainable development of the OFD ecosystem in Colombia and comparable Latin American markets.

### 6.2. Limitations and Recommendations for Future Research

Several limitations should be acknowledged. First, the cross-sectional design precludes causal inference, and the dependent construct examined was continuance intention, which, although widely recognized as a proximal antecedent of behavior, may not fully reflect actual retention because stated intentions can diverge from realized actions. Second, because all measures were self-reported by the same respondents, common method variance remains a potential concern. Although the multidimensional theoretical model clearly outperformed the single-factor specification and the results were further examined using a common latent factor sensitivity model, the absence of a theoretically unrelated marker variable prevented a more definitive marker-variable assessment. Finally, the subgroup analyses conducted across age, gender, and OFD experience were intended as robustness checks rather than formal tests of measurement or structural invariance. Future research should address these limitations by adopting longitudinal or multi-source designs, incorporating marker variables into survey instruments, linking intentions with observed behavioral outcomes, and conducting formal multigroup CFA and structural invariance analyses across relevant demographic and behavioral segments.

The combined online and interviewer-assisted recruitment mitigates but does not eliminate self-selection, and the sample skews toward urban, digitally experienced, and well-educated users, constraining generalizability to lower-income, rural, and infrequent users. Two choices warrant refinement: the eligibility criterion, namely use within the previous three months, is permissive and admits infrequent users, so stricter habitual-use thresholds would sharpen the focus on continuance, and income, operationalized through absolute peso brackets, would be better captured by measures adjusted for household size, dependents, and purchasing power.

From a measurement standpoint, the average variance extracted for Delivery Trust was marginally below the 0.50 threshold before rounding; though retained on the basis of acceptable reliability and significant loadings, its indicators merit refinement. In particular, Delivery Trust would benefit from an expanded indicator set that distinguishes instrumental trust—order accuracy and punctuality—from relational trust—effective recovery and platform accountability—which should raise its average variance extracted while sharpening its theoretical precision. Future research should undertake formal multigroup invariance testing across segments and model the mediating mechanisms, such as trust and quality operating through satisfaction, omitted from the present direct-effects specification. Cross-national designs (e.g., Brazil, Mexico, Chile, and Peru) and longitudinal studies would strengthen external validity and clarify how continuance evolves as perceptions accumulate, while qualitative methods would enrich interpretation of how fulfillment failures and food-quality disappointments shape continuance.

## Figures and Tables

**Table 1:** Theoretical contributions of the integrated frameworks to the LAE-UTAUT2 model.

Theoretical Foundation	Core Premise	Contribution to LAE-UTAUT2	Function in Explaining OFD Continuance
UTAUT2 [6]; UTAUT [25]	Integrative model of consumer technology acceptance and use.	Performance Expectancy, Effort Expectancy, Social Influence, Facilitating Conditions, Hedonic Motivation.	Technological-motivational backbone; utilitarian and hedonic antecedents of intention.
ECT [7,26]	Continuance is driven by satisfaction and the confirmation of expectations after use.	Reframes the dependent construct as continuance intention.	Converts adoption beliefs into post-experience continuance judgments.
Trust in e-commerce [11,21,23]	Trust mitigates uncertainty and perceived risk in digital exchange.	Delivery Trust (DT).	Relational determinant capturing fulfillment reliability under institutional risk.
SERVQUAL [15]; IS Success [14]	Quality as the gap between expected and perceived performance.	Food Quality Perception (FQ).	Outcome-quality determinant unique to food-bearing logistics.
Theory of Planned Behavior [8]	Intention reflects attitudes, subjective norms, and perceived control.	Complementary grounding for SI, FC, and HM.	Behavioral-intention scaffolding for the antecedents.
Diffusion of Innovations [30]	Adoption depends on relative advantage, compatibility, and complexity.	Complementary grounding.	Situates continuance within innovation-adoption perceptions.

**Table 2:** Demographic characteristics.

Variable	Category	*n*	%
Gender	Female	1134	53.2
	Male	906	42.5
	Prefer not to say	47	2.2
	Non-binary/Other	43	2
Age	18–24 years	614	28.8
	25–34 years	813	38.2
	35–44 years	395	18.5
	45–54 years	211	9.9
	55 years or older	97	4.6
City	Bogota	786	36.9
	Medellin	493	23.1
	Cali	348	16.3
	Barranquilla	234	11
	Other	269	12.6
Educational level	High school or less	272	12.8
	Technical/technological degree	491	23.1
	Undergraduate degree	862	40.5
	Postgraduate degree	505	23.7
Employment status	Employed	825	38.7
	Student	519	24.4
	Self-employed/freelance	419	19.7
	Unemployed	254	11.9
	Other	113	5.3
Monthly income (COP)	Less than $1,000,000	477	22.4
	$1,000,001–$2,000,000	685	32.2
	$2,000,001–$4,000,000	568	26.7
	More than $4,000,000	400	18.8
Main OFD app	Rappi	995	46.7
	More than one	453	21.3
	PedidosYa	384	18
	Uber Eats	298	14
Frequency of use	Less than once a month	229	10.8
	1–3 times per month	579	27.2
	Once per week	577	27.1
	Several times per week	552	25.9
	Daily	193	9.1
Experience using OFD apps	Less than 6 months	176	8.3
	6–12 months	292	13.7
	1–3 years	825	38.7
	More than 3 years	837	39.3

**Table 3:** Descriptive Statistics and Reliability Coefficients.

Construct	Items	Mean	SD	Cronbach’s Alpha
Performance Expectancy (PE)	PE1, PE2, PE3, PE4	5.566	0.874	0.809
Effort Expectancy (EE)	EE1, EE2, EE3, EE4	5.84	0.793	0.824
Social Influence (SI)	SI1, SI2, SI3	4.748	1.082	0.782
Facilitating Conditions (FC)	FC1, FC2, FC3	5.548	0.899	0.807
Hedonic Motivation (HM)	HM1, HM2, HM3	5.18	1.021	0.763
Delivery Trust (DT)	DT1, DT2, DT3, DT4	4.702	1.049	0.799
Food Quality Perception (FQ)	FQ1, FQ2, FQ3	4.656	1.024	0.791
Continuance Intention (CI)	CI1, CI2, CI3, CI4	5.298	0.982	0.833

**Table 4:** CFA measurement model: factor loadings, CR, and AVE.

Construct	Item	Std. Loading	*p*	Cronbach Alpha	CR	AVE
PE	PE1	0.721	-	0.809	0.809	0.515
	PE2	0.703	<0.001			
	PE3	0.722	<0.001			
	PE4	0.723	<0.001			
EE	EE1	0.754	-	0.824	0.824	0.54
	EE2	0.751	<0.001			
	EE3	0.69	<0.001			
	EE4	0.743	<0.001			
SI	SI1	0.745	-	0.782	0.782	0.545
	SI2	0.726	<0.001			
	SI3	0.742	<0.001			
FC	FC1	0.759	-	0.807	0.807	0.583
	FC2	0.767	<0.001			
	FC3	0.763	<0.001			
HM	HM1	0.723	-	0.763	0.763	0.518
	HM2	0.717	<0.001			
	HM3	0.72	<0.001			
DT	DT1	0.705	-	0.799	0.799	0.499
	DT2	0.704	<0.001			
	DT3	0.696	<0.001			
	DT4	0.72	<0.001			
FQ	FQ1	0.763	-	0.791	0.791	0.558
	FQ2	0.737	<0.001			
	FQ3	0.742	<0.001			
CI	CI1	0.731	-	0.833	0.833	0.556
	CI2	0.751	<0.001			
	CI3	0.744	<0.001			
	CI4	0.756	<0.001			

**Table 5:** Discriminant validity: HTMT matrix.

	PE	EE	SI	FC	HM	DT	FQ	CI
PE	1							
EE	0.504	1						
SI	0.387	0.316	1					
FC	0.486	0.559	0.351	1				
HM	0.493	0.405	0.406	0.346	1			
DT	0.426	0.401	0.331	0.406	0.37	1		
FQ	0.358	0.27	0.271	0.275	0.421	0.461	1	
CI	0.624	0.461	0.458	0.431	0.555	0.508	0.492	1

**Table 6:** Model fit and common method bias assessment.

Model	Chi-Square	df	CFI	TLI	RMSEA	AIC	BIC
Measurement model (CFA)	367.318	322	0.998	0.998	0.008	167.655	643.421
Structural model (SEM)	367.317	322	0.998	0.998	0.008	167.655	643.421
Single-factor model (CMB check)	9551.453	350	0.59	0.557	0.111	103.031	420.209

Note. The measurement (CFA) and structural (SEM) models are statistically equivalent because the structural component is recursive and saturated—all paths to CI are estimated and the predictors are freely correlated—so identical fit values are expected by construction and do not result from correlated-error respecification.

**Table 7:** SEM structural path estimates and hypothesis testing.

Hypothesis	Path	Unstd. Estimate	Std. Beta	*p*	Decision	f^2^
H1	PE -> CI	0.343	0.311	<0.001	Supported	0.084
H2	EE -> CI	0.081	0.068	0.017	Supported	0.000
H3	SI -> CI	0.127	0.138	<0.001	Supported	0.017
H4	FC -> CI	0.026	0.024	0.398	Not supported	0.000
H5	HM -> CI	0.177	0.181	<0.001	Supported	0.020
H6	DT -> CI	0.137	0.145	<0.001	Supported	0.014
H7	FQ -> CI	0.165	0.176	<0.001	Supported	0.025

**Table 8:** SEM robustness model with control variables.

Predictor	Unstandardized Estimate	β	*p*-Value	Result
PE	0.322	0.292	<0.001	Significant
EE	0.094	0.079	0.005	Significant
SI	0.128	0.139	<0.001	Significant
FC	0.021	0.019	0.487	Not significant
HM	0.186	0.190	<0.001	Significant
DT	0.131	0.139	<0.001	Significant
FQ	0.172	0.185	<0.001	Significant
Gender: male	−0.042	−0.024	0.193	Not significant
Gender: other/prefer not to say	−0.047	−0.011	0.548	Not significant
Age	0.011	0.014	0.419	Not significant
Educational level	−0.017	−0.019	0.292	Not significant
Income level	0.017	0.020	0.264	Not significant
Frequency of use	0.104	0.137	<0.001	Significant
Experience using OFD apps	−0.007	−0.008	0.666	Not significant

β = standardized path coefficient. Model fit: CFI = 0.986, TLI = 0.984, RMSEA = 0.017.

## Data Availability

The original data presented in this study are openly available at https://docs.google.com/spreadsheets/d/1x-kRMf7uis1ITtfiC0mSbI5pcOoKwCfH/edit?usp=sharing&ouid=106903503594857624193&rtpof=true&sd=true (accessed on 2 December 2025).
